# Natural Compounds: A Potential Treatment for Alcoholic Liver Disease?

**DOI:** 10.3389/fphar.2021.694475

**Published:** 2021-07-05

**Authors:** Junbin Yan, Yunmeng Nie, Minmin Luo, Zhiyun Chen, Beihui He

**Affiliations:** ^1^ The First Affiliated Hospital of Zhejiang Chinese Medical University, Hangzhou, China; ^2^ Key Laboratory of Integrative Chinese and Western Medicine for the Diagnosis and Treatment of Circulatory Diseases of Zhejiang Province, The First Affiliated Hospital of Zhejiang Chinese Medical University, Hangzhou, China

**Keywords:** alcoholic liver disease, natural compounds, oxidative stress, inflammatory pathway, autophagy, apoptosis

## Abstract

Excessive alcohol intake is a direct cause of alcoholic liver disease (ALD). ALD usually manifests as fatty liver in the initial stage and then develops into alcoholic hepatitis (ASH), fibrosis and cirrhosis. Severe alcoholism induces extensive hepatocyte death, liver failure, and even hepatocellular carcinoma (HCC). Currently, there are few effective clinical means to treat ALD, except for abstinence. Natural compounds are a class of compounds extracted from herbs with an explicit chemical structure. Several natural compounds, such as silymarin, quercetin, hesperidin, and berberine, have been shown to have curative effects on ALD without side effects. In this review, we pay particular attention to natural compounds and developing clinical drugs based on natural compounds for ALD, with the aim of providing a potential treatment for ALD.

## Introduction

Alcohol liver disease (ALD), a liver disease caused by long-term heavy alcohol intake, is the leading cause of chronic liver disease (CLD), especially in Europe and America (Hosseini et al., 2019). With economic development, the average daily alcohol intake of people in developing areas is gradually increasing, accompanied by an increasing ALD prevalence (Mukherjee et al., 2017; Wang et al., 2019; Mitra et al., 2020). Alcoholism causes nearly 2.5 million deaths annually, accounting for 4% of all deaths. Most mortality from alcoholism is caused by ALD (Jaurigue and Cappell, 2014). ALD is gradually becoming a worldwide public health problem.

Few specific medicines for ALD treatment exist. The development of effective and safer ALD treatments is an unmet clinical need (Singal et al., 2018). In China, herbs have been widely used to treat diseases for more than a thousand years. However, because of their complex composition, herbs cannot be used globally. Natural compounds in herbs are active ingredients with defined chemical compositions, and their curative effect has progressively been recognised (Zeng H. et al., 2019). Currently, various studies have verified the efficacy of numerous natural compounds on ALD. This review attempts to summarise and discuss the current research on ALD treatment with natural compounds, promoting the use of herbs in treating ALD.

## Mechanisms of Alcoholic Liver Disease Pathogenesis

### Disordered Lipid Metabolism

**FIGURE 1:**
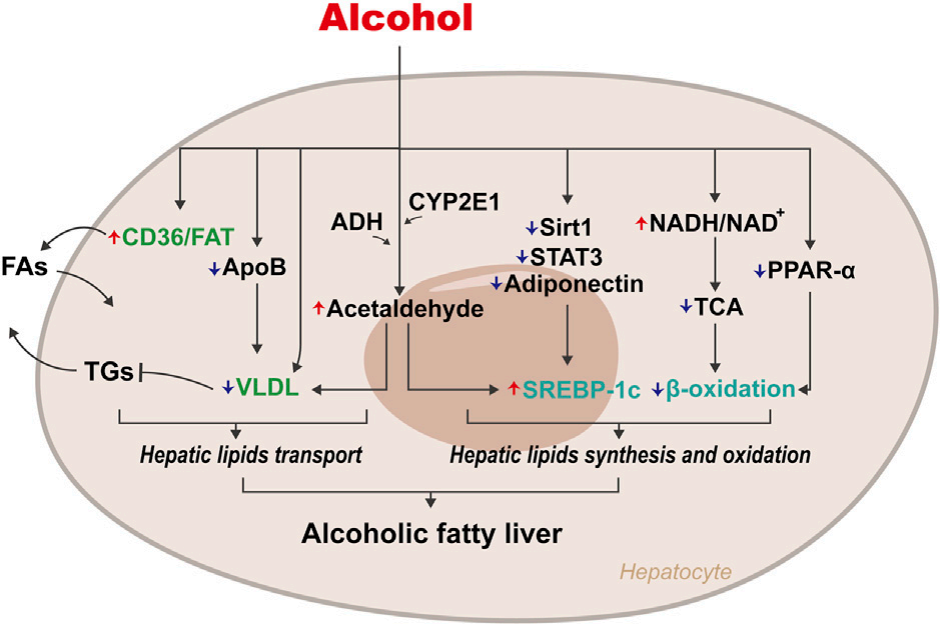
Mechanisms of disordered lipid metabolism on Alcoholic fatty liver. The abnormal accumulation of hepatic lipids caused by alcohol is mainly due to disorders of lipids transport and lipids synthesis and oxidation. Periodical alcohol intake upregulates CD36/FAT expression, leading to excessive intake of lipids, while inhibiting the synthesis of ApoB and VLDL, leading to the disorder of lipids excretion. Excessive alcohol intake increases the ratio of NADH/NAD^+^, which in turn inhibits β-oxidation of lipids. Alcohol can also promote lipids synthesis by up-regulating the expression of SREBP-1c.

ALD is always accompanied by hepatic steatosis induced by the imbalance of lipid metabolism originating from excess alcohol (Figure 1). CD36/FA transportase (FAT), a vital fatty acid (FA) transporter, plays a crucial role in FA uptake: upregulating the expression of CD36/FAT will promote the uptake of FAs by the liver (Febbraio et al., 2001; He et al., 2011). Animal experiments found that sustained alcohol intake upregulated the expression of CD36/FAT, causing excessive intake of FAs in mice and rats and resulting in hepatic steatosis (Ronis et al., 2011; Clugston et al., 2014). Very low-density lipoprotein (VLDL), the main form of endogenous triglyceride (TG) transport, contributes to the release of lipids in the liver. Excess alcohol intake will inhibit apolipoprotein B (ApoB) and VLDL synthesis, resulting in the inability to smoothly release hepatic lipids, resulting in steatosis (Tomita et al., 2004). Acetaldehyde, the intermediate product of alcohol, can also cause a decrease in VLDL synthesis by destroying the structure and function of Golgi apparatus, which in turn leads to lipid accumulation in the liver.

Early studies also showed that excessive intake of alcohol increases the ratio of reduced nicotinamide adenine dinucleotide (NADH)/oxidised nicotinamide adenine dinucleotide (NAD^+^) in hepatocytes, which in turn inhibits the tricarboxylic acid (TCA) cycle, interferes with β-oxidation of FAs, and results in excessive accumulation of FAs (You and Arteel, 2019). Simultaneously, the increase in NADH directly promotes the synthesis of FAs so that excessive lipids accumulate in hepatocytes. Alcohol also inhibits β-oxidation of FAs in hepatocytes by inhibiting peroxisome proliferator-activated receptor α (PPARα) (Wagner et al., 2011).

Additionally, cytochrome P450 2E1 (CYP2E1), an enzyme found in both the endoplasmic reticulum (ER) and mitochondria of hepatocytes, could metabolise alcohol to acetaldehyde in the presence of oxygen (Lieber 2004). Acetaldehyde directly upregulates the expression of sterol regulatory element-binding protein-1c (SREBP-1c) to promote TG synthesis (You et al., 2002). Ji et al. found that destroying SREBP-1c in mice improved alcohol-induced fatty liver, thus confirming the critical role of SREBP-1c in the pathogenesis of ALD (Ji et al., 2006). Alcohol also directly upregulates SREBP-1c expression to cause liver steatosis by inhibiting genes regulating SREBP-1c, such as Sirtuin1 (Sirt1), adiponectin, and signal transducer and activator of transcription 3 (STAT3) (Horiguchi et al., 2008; You et al., 2008; You and Rogers, 2009).

### Excessive Oxidative Stress

The liver, a vital organ for alcohol metabolism, can be damaged by by-products of alcohol decomposition, such as reactive oxygen species (ROS) (D'Autréaux and Toledano, 2007). CYP2E1 metabolises alcohol and produces ROS, which is closely associated with ALD development. Butura et al. found that the expression of oxidative stress-related genes and alcohol-induced hepatic injuries in mice were increased in the presence of the CYP2E1 transgene (Butura et al., 2009). Upregulation of CYP2E1 expression by adenovirus-mediated gene transfer in mice also promoted oxidative stress and liver damage (Bai and Cederbaum, 2006). By constructing a HepG2 cell line overexpressing CYP2E1, Wu et al. proved that CYP2E1 could inhibit the expression of antioxidant genes, such as glutathione (GSH), catalase (CAT), microsomal glutathione transferase 1 (MGST1), and α-glutathione S-transferase (α-GST), and facilitate alcohol to induce oxidative stress (Wu and Cederbaum, 2005).

Acetaldehyde is linked to the pathway by which alcohol causes oxidative stress in the liver. Acetaldehyde is highly reactive and toxic, and causes structural and functional damages to organelles such as mitochondria (Seitz and Stickel, 2007). The functional impairment of mitochondria will decrease ATP produced by the respiratory chain and increase ROS generation (O'Shea et al., 2010a). Overproduction of ROS causes oxidative stress, therefore leading liver damage (Cichoż-Lach and Michalak, 2014).

### Abnormal Inflammation

Alcohol-induced sensitisation of Kupffer cells (KCs) to lipopolysaccharide (LPS) is considered a hallmark of inflammation and ALD. The level of circulating LPS in ALD patients is significantly elevated and increases with disease severity (Fujimoto et al., 2000). The secretion of pro-inflammatory cytokines, such as chemokines (IL-8 and CCL2), interleukins (IL-1β and IL-6), and tumour necrosis factor (TNF), will be activated after excess LPS is recognised by Toll-like receptor 4 (TLR4), the vital LPS receptor (McClain and Cohen, 1989; Massey and Arteel, 2012; Roy et al., 2015). The above pro-inflammatory cytokines subsequently cause inflammation and hepatic injury. TNF, for instance, is a crucial mediator of alcohol-induced hepatic damage (Ajakaiye et al., 2011). The upregulation of TNF causes liver inflammation and inhibits FA β-oxidation (Mandrekar and Szabo, 2009). Continuous intake of excessive alcohol may promote TLR4 in KCs to recognise LPS, leading to the secretion of pro-inflammatory cytokines and further liver inflammation and ALD development.

The polarisation of KCs is also essential for the progression of inflammation (Xu et al., 2013; Zeng et al., 2016). Polarised KCs can be classified into two phenotypes, M1 and M2. M1 macrophages are inflammatory. They are always polarised by LPS alone or in the presence of Th1 cytokines (such as IFN-γ and GM-CSF), and they secrete pro-inflammatory cytokines such as IL-1β, IL-6, IL-12, IL-23, and TNF-α. M2 macrophages polarised by Th2 cytokines such as IL-4 and IL-13 have anti-inflammatory effects and produce anti-inflammatory cytokines such as IL-10 and TGF-β (Shapouri-Moghaddam et al., 2018). Promoting the polarisation of anti-inflammatory M2 KCs will effectively prevent alcohol-induced steatosis and apoptosis of hepatocytes (Louvet et al., 2011). Wan et al. showed that the expression of M2-related genes (CD206 and CD163) in the liver of ALD patients with excessive alcohol intake was significantly reduced. After treatment, BALB/c mice with ALD showed superior M2 KC polarisation. Experimental results indicated that alcohol causes inflammation and ALD by inhibiting the preferential polarisation of KCs to M2. M2-induced apoptosis of M1 KCs may be a novel strategy to improve alcohol-induced inflammatory hepatic damage (Wan et al., 2014).

### Programmed Cell Death

Autophagy, a cellular self-protection program, is responsible for maintaining cell homeostasis by routing cytoplasmic macromolecules or damaged organelles to lysosomes for degradation (Ueno and Komatsu, 2017; Yu et al., 2018). Selective autophagy mediates the degradation of specific cargo, such as mitochondria and lipid droplets (LDs) for self-protection (Schulze et al., 2017; Gatica et al., 2018). Interestingly, alcohol has a two-way regulatory effect on autophagy (both activating and inhibitory). Administration of alcohol activates autophagy in primary cultured mouse hepatocytes and hepatoma cells that express ADH and CYP2E1, as well as in the liver of acute alcohol binge-treated mice and mitigates alcohol-induced hepatotoxicity by spot clearing damaged mitochondria and accumulated LDs (Ding et al., 2010). A diet with a low alcohol content (accounting for 29% of the caloric intake) promotes autophagy in mice. Nevertheless, when the alcohol level is higher (accounting for 36% of the caloric intake), autophagic flux will be inhibited. Menk et al. found that long-term alcohol feeding in rats increased the protein level of p62 (Menk et al., 2018). Thomes et al. also proved that the expression of p62 protein in alcohol-fed mice increased along with decreased lysosomes and levels of autophagosome-lysosome fusion (Thomes et al., 2015). The above experiments have proven that autophagy is inhibited by excessive alcohol intake, which leads to lipid accumulation and severe oxidative stress and further accelerates alcoholic liver injury.

Apoptosis is the process by which cells actively end their lives to maintain organismal homeostasis. Apoptosis is an important pathological feature of ALD (Nagy et al., 2016). Alcohol can cause hepatocyte apoptosis by activating caspase-dependent/caspase-independent apoptotic pathways and ER stress. Petrasek et al. indicated that the STING-IRF3 pathway is a critical determinant of ALD, which links ER stress with hepatocyte apoptosis (Petrasek et al., 2013). Alcohol induces ER stress and triggers the binding of IRF3 to stimulator of interferon gene (STING) and subsequent activation of IRF3. Phosphorylated IRF3 activates the pro-apoptotic molecule B-cell lymphoma 2 (Bcl2)-associated X protein (Bax) and leads to hepatocyte apoptosis. The lack of STING will prevent alcohol-induced phosphorylation of IRF3 and subsequent hepatocyte apoptosis. Using loss- and gain-of-function mutations for transglutaminase 2 (TG2) and Sp1 transcription factor (Sp1) *in vitro* and *in vivo*, Tatsukawa et al. showed that TG2 is a vital cross-linking enzyme in alcohol-induced hepatocyte apoptosis (Tatsukawa et al., 2009). Compared with normal mice, TG2^−/−^ mice showed significantly reduced hepatocyte apoptosis after alcohol administration. It has also been proven that in AS mice and patients with AS, TG2 translocates into the hepatocyte nucleus and inhibits the activity of Sp1, leading to the downregulation of hepatocyte growth factor receptor, cellular-mesenchymal epithelial transition factor (c-Met), and apoptosis (Tatsukawa and Kojima, 2010). The above-mentioned caspase-independent pro-apoptotic pathway is novel and deserves more attention.

### Intestinal Microbes and Intestine-Liver Axis

The gut is the physiological habitat for various microbes, such as bacteria, archaea, fungi, yeast, and viruses, which carry more than three million unique genes (Guarner and Malagelada, 2003). Intestinal microbes are involved in the synthesis of amino acids, the catabolism of bile acids (BAs), the provision of energy, and intestinal barrier preservation and can better regulate alcohol-related hepatic damage, which is closely related to the development of ALD (Bajaj 2019). Researchers have regarded the intestinal microbiome as a viable target for ALD treatment (Sarin et al., 2019).

Alcohol abuse promotes the overgrowth of intestinal bacteria and causes changes in intestinal microbial composition (Bjørkhaug et al., 2019). Yan et al. constructed an ALD disease model by feeding C57/B6 mice intragastric ethanol for three weeks. The composition of intestinal microbes in ALD mice changed (Lactococcus, Pediococcus, *Lactobacillus*, Lactobacillales decreased, while Verrucomicrobia and Bacteroidetes significantly increased), and the expression of regenerating family member 3 gamma (Reg3γ) was inhibited. Prebiotic fructooligosaccharide (FOS) treatment effectively upregulated the expression of Reg3γ, reduced intestinal bacterial overgrowth, and improved hepatic inflammation in ALD mice (Yan et al., 2011). Antifungal treatment improves liver damage caused by alcohol. Mutlu et al. directly proved that transplantation of probiotic *Lactobacillus* strains could improve liver damage in ALD mice (Mutlu et al., 2009). Recent studies have also shown that changes in the composition and metabolites of intestinal microbes may affect brain function and behaviour (Hsiao et al., 2013). Changes in the intestinal microbes caused by alcohol may influence physical activity and cognitive ability, enhancing the tendency to drink, further causing a vicious cycle of alcoholic liver injury (Leclercq et al., 2019).

Intestine-liver axis is involved in the initiation of inflammation in ALD. Alcohol intake destroys the intestinal barrier and increases intestinal permeability, causing bacterial (such as LPS) translocation in ALD patients and ALD experimental models (Zhou and Zhong, 2017). Under normal circumstances, the level of LPS in blood is extremely low and does not cause significant responses. However, because alcohol change intestinal permeability, a large amount of LPS reaches the liver through the portal vein, activates the innate immune response, and causes inflammation, leading to ALD deterioration (Arab et al., 2018).

**FIGURE 2:**
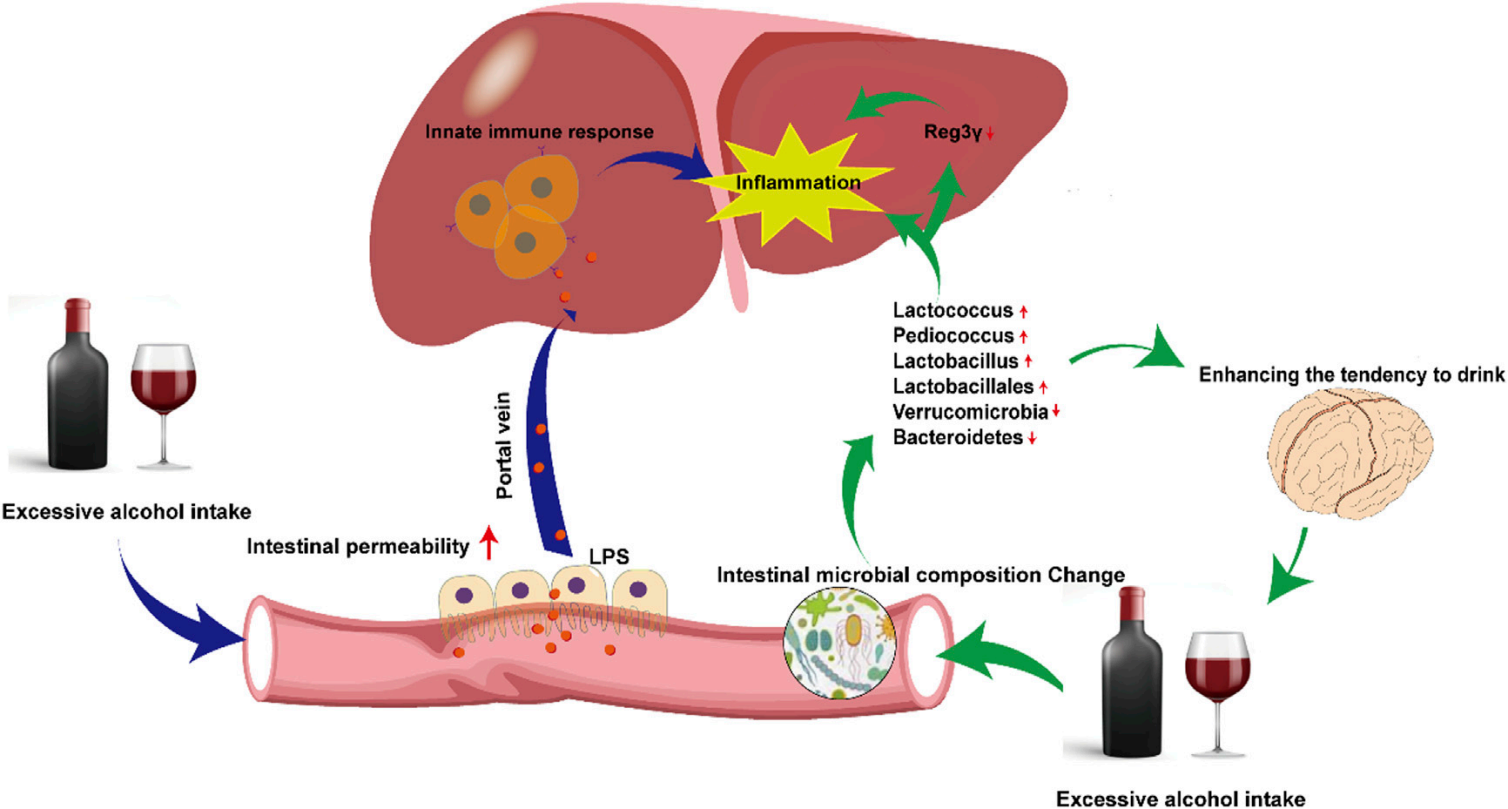
Mechanisms of disordered intestinal microbes and intestine-liver axis on Alcoholic fatty liver. Excessive alcohol consumption alters the distribution of intestinal microbes and permeability. Changes in intestinal microbes directly cause hepatic inflammation, also indirectly aggravate inflammation by downregulating the expression of Reg3γ. Alterations in intestinal permeability leads to more LPS entering the liver via portal vein, activating innate immunity and aggravating hepatic inflammation.

The possible mechanism linking intestinal microbes and intestine-liver axis to ALD is depicted in Figure 2.

## Current State of Alcoholic Liver Disease Drug Treatment

Glucocorticoids are always used to treat severe alcoholic hepatitis (SAH) and acute alcoholic hepatitis (AAH). The potential therapeutic mechanism of glucocorticoids is to suppress inflammation by reducing immune and pro-inflammatory cytokine responses (Taïeb et al., 2000). However, glucocorticoids have many side effects. Long-term use will increase the risk of complications such as infections, gastrointestinal bleeding, and even kidney failure (Naveau et al., 2004). Moreover, the use of glucocorticoids has many contraindications, such as sepsis, hepatitis B, hepatorenal syndrome, etc. Therefore, its indication is relatively limited (Lucey et al., 2009).

Pentoxifylline (PTX) is an oral phosphodiesterase inhibitor that can suppress inflammation by reducing serum TNF-α levels (Doherty et al., 1991). PTX can be used for a short period in patients with AH when glucocorticoids are counter indicated (Akriviadis et al., 2000; O'Shea et al., 2010b). However, the study found that the efficacy of PTX alone or PTX combined with glucocorticoids in the treatment of AH was not better than that of glucocorticoids alone. The randomised clinical trial results of Whitfield, Kate et al. did not support that PTX has a positive effect on AH participants (Whitfield et al., 2009).

Metadoxine, a drug used to treat acute alcoholism, could accelerate ethanol excretion and lower the level of ethanol in the blood (Vonghia et al., 2008). However, Mao Yi-min et al. found that, among subjects who were treated with metadoxine, the total effective rate of liver function improvement in patients who quit drinking during the study period was better than that in patients who did not stop drinking (Mao et al., 2009). The results showed that metadoxine may improve liver function indirectly by improving alcohol excretion, but it cannot directly improve liver steatosis or inflammation.

PTX alone has poor efficacy and is always combined with glucocorticoids in treating acute alcoholic hepatitis or severe alcoholic hepatitis, which is the critical stage of ALD. However, glucocorticoids have significant side effects and are not suitable for long-term treatment. Metadoxine is commonly used clinically for acute alcoholism because it promotes alcohol excretion. However, all of them indirectly improved liver damage caused by long-term drinking by improving alcohol metabolism. Metadoxine is not a specific treatment for ALD per se.

## Natural Compounds and Their Mechanisms Action in the Treatment of Alcoholic Liver Disease

**FIGURE 3:**
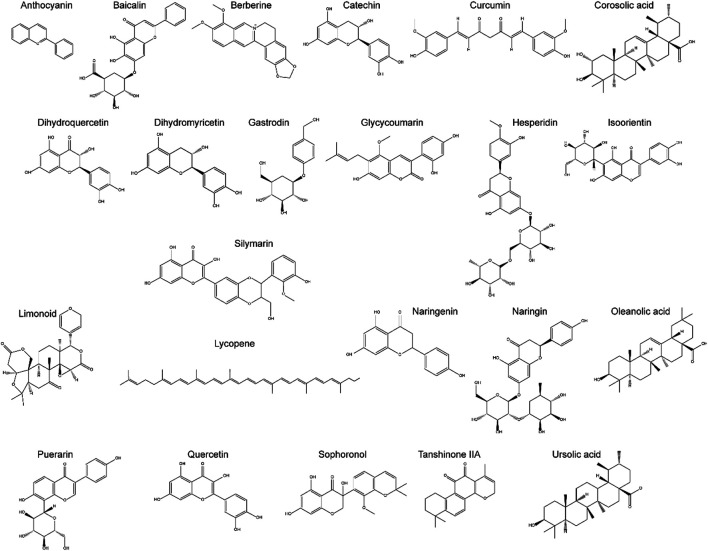
The chemical structure of Natural components for the potential treatment of ALD.

Increasing evidence indicates that natural compounds can effectively inhibit ALD progression. Natural compounds have been demonstrated to be effective towards ALD by engaging in a variety of pathways, including improvement of lipid metabolism, suppression of oxidative stress and inflammation, and reduction of programmed cell death (PCD). Natural compounds affect cellular functions broadly with few side effects, and can be administered at different stages of ALD. Table 1 summarizes the effects and the mechanisms of action of 23 natural compounds on ALD from recent studies. Figure 3 shows the chemical structure of these natural compounds. We will introduce these natural compounds in the following sections according to their mechanisms of action.

**TABLE 1:** **|** Natural compound derived from herbs for the potential treatment for ALD.

Natural components	CAS	MF	MW (g/mol)	Mechanisms	References
Anthocyanin	14,051–53-7	C_15_H_11_O^+^	207.25	Improve inflammation	Jiang, et al. (2016)
Baicalin	21,967–41-9	C_21_H_18_O_11_	446.4	Inhibit TLR4-mediated inflammation	Kim, and Lee, (2012)
Berberine	2,086–83-1	C_20_H_18_NO_4_^+^	336.4	Adjust intestinal microbes and improve inflammation	Li, et al. (2020)
Catechin	7,295–85-4	C_15_H_14_O_6_	290.27	Inhibit the activation of NF-κB and prevent necrotizing inflammatory changes	Bharrhan, et al. (2011)
Corosolic acid	4,547–24-4	C_30_H_48_O_4_	472.7	Activate autophagy	Guo, et al. (2016)
Curcumin	458–37-7	C_21_H_20_O_6_	368.4	Reduce hepatic lipid accumulation	Lu, et al. (2015)
Dihydromyricetin	27,200–12-0	C_15_H_12_O_8_	320.25	Improve oxidative stress and lipid metabolism	(Silva, et al. (2021); Silva, et al. (2020))
Dihydroquercetin	480–18-2	C_15_H_12_O_7_	304.25	Reduce lipid accumulation by inhibiting lipid synthesis and promoting lipid β-oxidation	Zhang, et al. (2018a)
Gastrodin	62,499–27-8	C_13_H_18_O_7_	286.28	Reduce alcohol-induced hepatic damage by inhibiting apoptosis and	Zhang, et al. (2018b)
Glycycoumarin	94,805–82-0	C_21_H_20_O_6_	368.4	Improve oxidative stress; Promote autophagy	Song, et al. (2015)
Hesperidin	520–26-3	C_28_H_34_O_15_	610.6	Improve lipid metabolism and oxidative stress	(Tabeshpour, et al. (2020); Zhou and Zhong, (2017))
Isoorientin	4,261–42-1	C_21_H_20_O_11_	448.4	Reduce oxidative stress by increasing the activities of antioxidant SOD and GSH-Px	Chen, et al. (2013a)
Limonoid	1,180–71-8	C_26_H_30_O_8_	470.53	Improve oxidative stress and inflammation	Valansa, et al. (2020)
Lycopene	502–65-8	C_40_H_56_	536.9	Improve oxidative stress; Regulates intestinal microbes	(Li, et al. (2018), Stice, et al. (2018))
Naringenin	67,604–48-2	C_15_H_12_O_5_	272.25	Improve lipid metabolism, oxidative stress, and reduce cell apoptosis	Lin, et al. (2017)
Naringin	10,236–47-2	C_27_H_32_O_14_	580.5	Reduce lipid accumulation, improve oxidative stress and inflammation	(Park, et al. (2013), Zhou, et al. (2019))
Oleanolic acid	508–02-1	C_30_H_48_O_3_	456.7	Improve oxidative stress	Liu, et al. (2014)
Puerarin	3,681–99-0	C_21_H_20_O_9_	416.4	Improve lipid metabolism by increasing the activity of ADH and ALDH	(Chen, et al. (2013b), Li, et al. (2013))
Quercetin	117–39-5	C_15_H_10_O_7_	302.23	Promote lipid autophagy; Regulate lipid peroxidation; Inhibit inflammation	(Zeng, et al. (2019b), Zhao, et al. (2021))
Silymarin	65,666–07-1	C_25_H_22_O_10_	482.4	Improve oxidative stress; Alleviating lipid peroxidation	(Song, et al. (2006), Tighe, et al. (2020), Zhang, et al. (2013))
Sophoronol	1,173,250–93-5	C_21_H_20_O_7_	384.4	Promote the activity ADH and ALDH; Improve inflammation	Choi, et al. (2009)
Tanshinone IIA	568–72-9	C_19_H_18_O_3_	294.3	Inhibit fatty acids synthesis and promote β-oxidation of fatty acids	Yin, et al. (2008)
Ursolic acid	77–52-1	C_30_H_48_O_3_	456.7	Reduce alcohol-induced hepatic damage by inhibiting apoptosis and	Ma, et al. (2020)

MW, molecular weight; MF, molecular formula.

### Natural Compounds That Improve Lipid Metabolism

Curcumin, the main active ingredient in turmeric, has been shown to reduce lipid accumulation in hepatocytes and subsequent steatosis by regulating the NF-E2–related factor 2 (Nrf2)-farnesoid X receptor (FXR) pathway (Lu et al., 2015). Some scholars also found that treatment with curcumin could suppress oxidative stress in ALD mice by reducing the generation of ROS (Rong et al., 2012).

Dihydroquercetin, one of the most common dihydroflavonoids, is widely found in milk thistles and onions. Zhang et al. proposed that dihydroquercetin improves alcoholic liver steatosis by regulating the activation of the SIRT1-AMPK pathway (Zhang et al., 2018a). Dihydroquercetin increased the activity of AMPK, thereby reducing the expression of SREBP1 in ethanol-treated HepG2 cells. Dihydroquercetin shows the ability to inhibit lipid formation and protect the liver, suggesting that dihydroquercetin has the potential as a treatment for ALD.

Tanshinone IIA is superabundant in the root of Salvia miltiorrhiza BUNGE. Yin et al. found that tanshinone IIA can inhibit the synthesis of FAs and stimulate the oxidation of FAs, improving ALD (Yin et al., 2008). Tanshinone IIA has a lipid-modulating effect and attenuates lipid accumulation by regulating LXRα/SREBP1 pathway in HepG2 Cells (Gao et al., 2021).

### Natural Compounds That Reduce Oxidative Stress

Silymarin, a flavonoid extracted from Silybum marianum, has been introduced as a hepatoprotective agent. Silymarin mainly repairs hepatic injury by alleviating lipid peroxidation and oxidative stress (Song et al., 2006; Tighe et al., 2020). Zhang, Wei et al. found that silymarin significantly increases the activities of superoxide dismutase (SOD) and glutathione peroxidase (GSH-Px) and reduces the level of malondialdehyde (MDA) in ALD rats, which are all closely related to the oxidative stress pathway (Zhang et al., 2013).

Isoorientin was confirmed to be abundant in buckwheat. In an animal experiment, Chen et al. discovered that isoorientin effectively increases the activities of the antioxidant SOD and GSH-Px and the expression of alpha-SMA, thereby suppressing alcohol-induced oxidative stress and protecting from liver fibrosis in Wistar rats (Chen X. et al., 2013). In addition, the antioxidant activity of isoorientin was compared with some antioxidants, including α-Tocopherol, ascorbic acid and other plant derived compounds. In short, most studies support isoorientin is a strong antioxidant (Watanabe 2007; Ziqubu et al., 2020).

Oleanolic acid exists abundantly in fruits, vegetables, and herbs. Oleanolic acid has been proven to have promising pharmacological activities, such as hepatoprotective effects and antioxidant activities (Tian et al., 2017; Lim et al., 2019). The antioxidant function of oleanolic acid may be related to the regulation of Nrf2 expression (Wang et al., 2013). Liu et al. found that oleanolic acid could protect rats against alcohol-induced hepatic damage by controlling Nrf2-related antioxidants to maintain redox balance (Liu et al., 2014).

### Natural Compounds That Suppress Inflammation

Anthocyanins are a group of plant pigments widely present in flowers, fruits, and cereals (Kong et al., 2003). The health promotion effect of anthocyanins has received increasing attention. Jiang et al. found that, after anthocyanin treatments, the expression of IFN-γ, TNF-a, TLR-4, VCAM-1, and CXCL-1 significantly decreased in ALD mice, suggesting that anthocyanin may suppress inflammation by downregulating proinflammatory cytokines (Jiang et al., 2016).

Baicalin, an active ingredient extracted from Scutellaria baicalensis, is widely used in herbal preparations to treat ALD and inflammation. According to recent studies, baicalin plays a beneficial role in various invasive toxicant models by inhibiting inflammatory responses (Lixuan et al., 2010; Guo et al., 2019). Additionally, Kim et al. directly confirmed that baicalin could reduce hepatocyte damage by inhibiting the TLR4-mediated inflammatory pathway in ALD rats (Kim and Lee, 2012).

Puerarin and sophoronol both increase the activity of ADH and ALDH, thereby promoting alcohol metabolism and reducing hepatotoxic damage caused by alcohol (Choi et al., 2009; Chen YX. et al., 2013). In addition, studies have shown that puerarin is able to treat ALD by inhibiting the expression of TNF-α, cyclooxygenase-2 (Cox-2), and 5-lipoxygenase (5-Lox) and other pro-inflammatory cytokines (Li et al., 2013).

Catechin, a polyphenol antioxidant, inhibits activation of NF-κB in rats upon excessive alcohol uptake and prevents necrotizing inflammatory changes (Bharrhan et al., 2011).

### Natural Compounds That Regulate Programmed Cell Death

Corosolic acid, also known as 2α-hydroxyursolic acid, is the main active ingredient found in banaba leaves. Corosolic acid has attracted considerable attention due to its anti-diabetic effect and was called “Phyto-insulin” (Sivakumar et al., 2009). As a potential activator of AMPK, corosolic acid can restore ethanol-suppressed autophagy via AMPK activation *in vivo* and *in vitro* (Guo et al., 2016).

Gastrodin is one of the main bioactive components of Gastrodia elata, an ancient Chinese medicinal plant, geranium. Zhang et al. showed that gastrodin significantly inhibited caspase-3 (CASP3) activation and apoptosis from ethanol-induced toxicity *in vivo* and *in vitro* (Zhang et al., 2018b).

Ursolic acid is a pentacyclic terpenoid carboxylic acid with many health functions, such as anti-oxidative, anti-inflammatory, anti-cancer, and hepato-protective activities (Jinhua, 2019). Ma et al. directly confirmed that ursolic acid reduced hepatocellular apoptosis and alleviated alcohol-induced liver injury via inhibition of CASP3 *in vivo* and *in vitro* (Ma et al., 2020)*.*

### Natural Compounds That Regulate Intestinal Microbes

Berberine, a quaternary ammonia compound derived from many herbs, has been widely used to treat hepatic injury. Li et al. found that berberine increased the abundance of the bacteria Akkermansia muciniphila and changed the overall gut microbial flora in ALD mice, helping maintain metabolic balance and reduce inflammation (Zhang et al., 2019; Li et al., 2020). Interestingly, berberine also activates a population of granulocytic myeloid-derived suppressor cell (G-MDSC)-like cells with an immune suppressive function in the liver of mice, thus alleviating alcohol-induced hepatic injury.

### Natural Compounds With Multiple Therapeutic Mechanisms

Naringenin, the main flavanone in pomelo, could effectively improve oxidative stress and inflammation, decrease hepatic damage. Lin et al. have further confirmed that it can treat the ALD of zebrafish larvae by enhancing oxidative stress and lipid metabolism and attenuating the hepatocyte apoptosis (Lin et al., 2017).

Naringin is a flavanone rich in pomelo. The administration of naringin effectively suppressed the production of pro-inflammatory cytokines in ALD mice (Park et al., 2013). In addition, naringin also attenuated hepatic injury in zebrafish with ALD by reducing lipid accumulation and oxidative stress (Zhou et al., 2019).

Hesperidin, a flavonoid compound with pharmacological activity, is widely found in citrus peels (Hemanth Kumar et al., 2017). Various experiments have shown that hesperidin can reduce oxidative stress and inflammation and treat hyperglycaemia and hyperlipidaemia with few side effects (Roohbakhsh et al., 2015; Li and Schluesener, 2017). Recently, some researchers have confirmed its efficacy in the treatment of ALD. Zhou et al. demonstrated that hesperidin could inhibit the accumulation of hepatic lipids by regulating the expression of lipid metabolism-related genes, thus protecting zebrafish larvae from steatosis after alcoholic exposure (Zhou et al., 2017).

Quercetin, commonly present in fruits and vegetables, alleviates oxidative stress, glutathione depletion, and pro-inflammatory cytokine production in ALD (Lee et al., 2019). Zeng et al. proved that quercetin activated AMPK and increased the colocalisation of hepatic LC3II and Perilipin 2 (PLIN2) to promote lipophagy, thereby improving the steatosis of mice with ALD (Zeng H. et al., 2019). Zhao et al. also confirmed that quercetin directly mitigates alcohol-induced hepatic steatosis in zebrafish via the regulation of the PI3K/Keap1/Nrf2 pathway (Zhao et al., 2021).

Dihydromyricetin, a bioflavonoid isolated from Hovenia dulcis, has been proven to improve hepatic lipid metabolism by reducing the NADPH/NAD + ratio (Silva et al., 2020). Silva et al. suggest that treatment with dihydromyricetin could repair the mitochondrial functions damaged by acetaldehyde toxicity and decrease the generation of ROS by controlling the AMPK/Sirt-1/PGC-1α pathway, thereby suppressing oxidative stress and improving the corresponding ALD (Silva et al., 2021).

Glycycoumarin, a representative coumarin in liquorice, has good bioavailability *in vivo*. Liquorice is a popular edible and medicinal plant widely used to treat various diseases, including liver diseases and inflammatory diseases (Li et al., 2019). It has been shown that glycycoumarin has a strong preventive effect on chronic and acute alcoholic liver injury in mice. Further mechanistic studies showed that the hepato-protective effect of glycycoumarin could be attributed to the activation of Nrf2 and autophagy (Song et al., 2015).

Lycopene is rich in tomatoes, watermelon, pink grapefruit, papaya, apricot, and guava (Crowe-White et al., 2019). Lycopene is best-known for its antioxidant potential but has many other functions, including regulating the immune system and metabolism (Wang, 2012). Lycopene increased the richness and diversity of intestinal bacteria, indicating that lycopene has a protective effect on intestinal microorganisms (Li et al., 2018). Stice et al. confirmed that lycopene could reduce oxidative stress by regulating CYP2E1 (Stice et al., 2018).

Limonoids are natural tetracyclic triterpenoids with a variety of biological properties (Fan et al., 2019). Previous studies have indicated anti-cancer, anti-viral, and hepato-protective activities of limonoids. Valansa et al. showed that limonin had a protective effect on ALD mainly through its antioxidant and anti-inflammatory properties (Valansa et al., 2020).

Overall, the toxicity and side effects of these natural compounds are low, but more clinical trials are needed. In addition, although natural compounds are easy to obtain and functions through multiple pathways in the treatment of ALD, some natural compounds, such as curcumin and silymarin, are easily eliminated from the body due to reduced bioavailability and fast metabolism. Thus, more sophisticated research is required to improve their pharmacokinetics. It is crucial to optimise the chemical structure of natural compounds and develop drugs with high bioavailability, such as curcumin phospholipid complexes.

## Clinical Progress of Natural Compounds

Despite much evidence supporting the effects of natural compounds on ALD, clinical trials are still limited and large-scale human studies may be needed to confirm these effects. In a clinical trial, Sasaki et al. found that Theracurmin, a highly absorptive curcumin dispersed with colloidal nanoparticles, could significantly decrease the concentration of acetaldehyde in the blood and reduce alcohol intoxication (Sasaki et al., 2011).

Silymarin consists of several ingredients such as silybin and silydianin, and silybin is the main active ingredient in silymarin, accounting for approximately 60% (Ha et al., 2019). Clinical studies have shown that silymarin can significantly reduce the levels of ALT, AST, and ALP in patients with ALD, and its effect is considerably superior to that of traditional hepato-protective agents (Saller et al., 2001; Saller et al., 2008). The results of combinatorial treatment with lamivudine in ALD patients with chronic hepatitis B showed that Silybum thistle could significantly reduce the levels of hyaluronic acid (HA), laminin (LN), type III collagen (PⅢP), and collagen IV (IVC), effectively improve liver functions and inhibit the development of hepatic fibrosis. Silymarin and its related nutrients or drugs have long been approved by the US Food and Drug Administration (FDA). Silymarin and its related herbal supplements are currently widely used in the clinic as liver protective agents characterised by their apparent hepato-protective effects and limited toxicity and side effects (Beyoğlu and Idle, 2020). Nearly a quarter of patients with liver diseases in the United States have been treated with silymarin and its related agents.

Among the 23 natural compounds mentioned above, silymarin is one of the few compounds commonly used in clinical practice. In contrast, other natural compounds have not been validated by clinical trials. Therefore, further in-depth studies are needed.

## Summary and Outlook

Natural compounds play a vital role in protecting human health and curing human diseases. Many drugs currently used in clinical practice are directly derived from or inspired by natural compounds. From the 1940s to 2010, 65% of anti-bacterial small molecules and 41% of anti-cancer small molecule drugs were derived from natural compounds and derivatives. Natural compounds are still a treasure trove of drug development and are the backbone of the modern drug development process.

ALD is a type of hepatic damage caused by excessive alcohol intake. The mechanism of liver injury caused by alcohol is complex and mainly involves the imbalance of lipid metabolism, liver inflammation, promotion of oxidative stress, and changes in the distribution of intestinal microbes. In the review, we summarised the current knowledge on 23 natural compounds whose efficacy against ALD has been confirmed through clinical studies or animal experiments. Compared with glucocorticoids and PTX, which are commonly used to treat AAH and SAH, natural compounds have the advantages of low toxicity and a broad range of applications. Among them, silymarin, hesperidin, curcumin, etc., have been proven to have excellent hepato-protective effects, are suitable treatment options by themselves or in combination with other drugs as a supplement.

However, natural compounds are not perfect, and they have obvious drawbacks. 1) The clinical efficacy of most natural compounds is limited due to their low bioavailability. 2) It is difficult to define the most appropriate drug form and dosage without standardised pharmaceutical technology. Thus, the optimal liver protection effect cannot be realised. 3) Most compounds have not been subjected to double-blind, placebo-controlled clinical trials to evaluate their efficacy and mechanisms of action of natural compounds are still unclear because of scarce studies.

**FIGURE 4:**
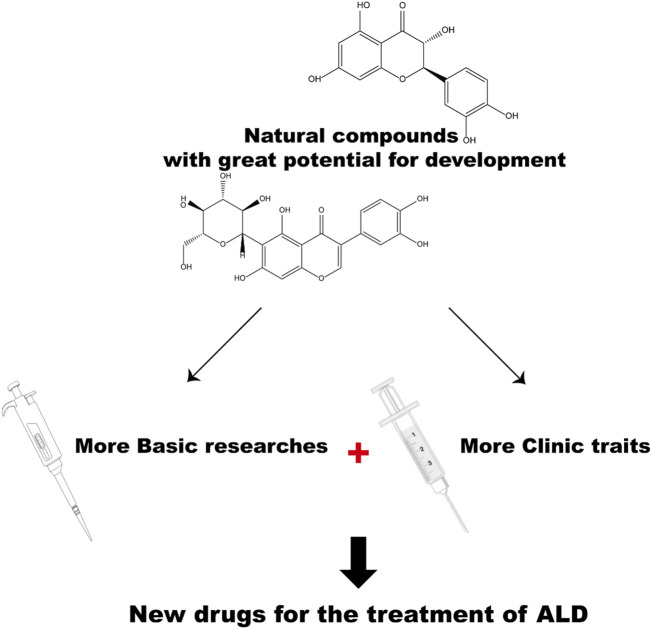
The prospects for developing natural compounds as ALD drugs.

Undoubtedly, natural compounds have enormous potentials to be developed as therapeutic drugs for ALD. However, in the field of ALD research, clinical trials are still needed for systematic reviews. Further research is needed on the potential role of natural compounds (Figure 4). We hope the review can provide theoretical support for the research on the mechanisms of ALD pathogenesis and progression, and subsequent therapeutic drug development for ALD.

## Glossary

**5-Lox**: 5-lipoxygenase
**α-GST**: α-glutathione S-transferase
**ADH**: alcohol dehydrogenase
**AFL**: alcoholic fatty liver
**ALD**: alcoholic liver disease
**ALDH2**: aldehyde dehydrogenase 2
**ApoB**: apolipoprotein B
**ASH**: alcoholic hepatitis
**BAs**: bile acids
**Bax**: B-cell lymphoma 2-associated X protein
**Bcl2**: B-cell lymphoma 2
**CASP3**: caspase-3
**CAT**: catalase
**CLD**: chronic liver disease
**c-Met**: cellular-mesenchymal epithelial transition factor
**Cox-2**: cyclooxygenase-2
**CYP2E1**: cytochrome P450 2E1
**ER**: endoplasmic reticulum
**FAs**: fatty acids
**FAT**: CD36/FA transportase
**FOS**: fructooligosaccharides
**FXR**: farnesoid X receptor
**GPx**: glutathione peroxidase
**GSH-Px**: glutathione peroxidase
**HCC**: hepatocellular carcinoma
KCs: kupffer cells
**LDs**: lipid droplets
**LIR**: LC3-interacting region
**LPS**: lipopolysaccharide
**MDA**: malondialdehyde
**MF**: molecular formula
**MGST1**: microsomal glutathione transferase 1
**MW**: molecular weight
**NAD+**: oxidized nicotinamide adenine dinucleotide
**NADH**: nicotinamide adenine dinucleotide
**NCLD**: noncirrhotic liver damage
**Nrf2**: NF-E2-related factor 2
**OPTN**: optineurin
**PCD**: programmed cell death
**PINK**: phosphatase and tensin homolog-induced kinase
**PLIN2**: perilipin 2
**PPARα**: peroxisome proliferator-activated receptor α
**PTEN**: phosphatase and tensin homolog
**Reg3g**: regenerating family member 3 gamma
**ROS**: reactive oxygen species
**Sirt1**: sirtuin1
**SOD**: superoxide dismutase: superoxide dismutase
**SOD**: superoxide dismutase: superoxide dismutase
**SREBP-1c**: sterol regulatory element-binding protein-1c
**STAT3**: signal transducer and activator of transcription 3
**STING**: stimulator of interferon gene
**TAC**: tricarboxylic acid cycle
**TCM**: traditional chinese medicine
**TG2**: transglutaminase 2
**TGs**: triglycerides
**TLRs**: toll-like receptors
**TNF**: tumour necrosis factor
**VLDL**: very low-density lipoprotein
